# Promising upshot of silver nanoparticles primed from *Gracilaria crassa* against bacterial pathogens

**DOI:** 10.1186/s13065-015-0120-5

**Published:** 2015-08-07

**Authors:** V Lavakumar, K Masilamani, V Ravichandiran, N Venkateshan, D V R Saigopal, C K Ashok Kumar, C Sowmya

**Affiliations:** Department of Pharmaceutical Biotechnology, Sree Vidhyanikethan College of Pharmacy, A.Rangampet, Tirupati, 517102 AP India; Faculty of Technology, University Malaysia Pahang, Lebuhraya Tun Razak, Gambang, 26300 Kuantan, Pahang Darul Makmur Malaysia; National Institute of Pharmaceutical Education and Research, Kolkata, 700032 WB India; Department of Pharmaceutical Chemistry, Sankarlingam Bhuvaneswari College of Pharmacy, Sivakasi, 626130 TN India; Department of Virology, S.V. University, Tirupati, 517502 AP India; Department of Pharmaceutics, Raghavendra Institute of Pharmaceutical Education and Research, Anantapuram, 515721 AP India

**Keywords:** Green synthesis, Red algae, *Gracilaria crassa*, Silver nanoparticles, Antibacterial activity

## Abstract

**Background:**

The study on newer antimicrobial agent from metal based nano materials has augmented in recent years for the management of multidrug resistance microorganisms. In our present investigation, we synthesized silver nanoparticles (AgNP’s) from red algae, *Gracilaria crassa* as beginning material which effectively condensed the silver ions to silver nanoparticles with less price tag and no risk.

**Methods:**

Silver nanoparticles were prepared by simple reaction of 1 mM AgNO_3_ with *G. crassa* extracts at room temperature. The fabricated AgNP’s were subjected for characterization and screened against various microorganisms for antibacterial activity.

**Results:**

UV–Vis spectroscopy (200–800 nm), XRD, FESEM and EDAX, were performed for AgNP’s. UV–Vis spectroscopy demonstrated the absorption edge at 443 nm and EDAX pattern is purely due to the particle size and face centered cubic (fcc) symmetry of nanoparticles. Average size lays at 122.7 nm and zeta potential was found to be −34.9 mV. The antibacterial outcome of synthesized AgNP’s (at the dose of 20 and 40 µg/ml) was evaluated against *Escherichia coli*, *Proteus mirabilis*, *Bacillus subtilis* and *Pseudomonas aeruginosa*. The mechanism of synthesized AgNP’s bactericidal bustle is discussed in terms of interaction with the cell membrane of bacteria. The activity was found to be sky-scraping in a dose dependent manner.

**Conclusion:**

Thus, environmental friendly, cost effective, non hazardous stable nanoparticles were prepared by green synthesis using red algae, *G. crassa*. Synthesized *G. crassa* AgNP’s were in acceptable size and shape. Further, it elicits better bactericidal activity against microorganism. This will assure the out put of superior antibacterial formulation for near future.

**Graphical Abstract:**

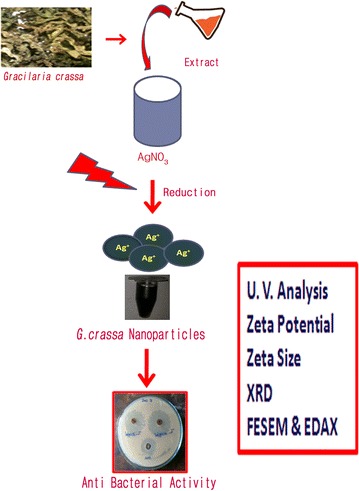

## Background

From ancient, handling of microbial infection is an exigent task for microbiologists. Countless drugs have been found to be successful, unfortunately, it leads to mount of drug resistance against particular pathogens with an outlook of stern issues in concern with public wellbeing [1]. Technical community is animatedly trying to expand groundbreaking concepts in drug delivery by challenging the new microbial agent with superior mode of action by its effectual target on the cell membrane or neither on intracellular targets [2, 3]. In 21st century, nanotechnology has become inevitable, not because of only claim and also by the way of synthesis. Two way synthesis like physical and chemical methods have several sizeable challenges like technically protracted, expensive and ecological hazardous defects [4]. The current art of exploration in research is heading towards green synthesis of high-yield, squat in cost, non-hazardous and eco-friendly metallic nanoparticles by plants and microorganisms [5, 6]. Due to hefty surface area, high reactivity and surface plasmon resonance, these nanoparticles were tailored for definite application by scheming into unambiguous size, shape and morphology leading to exhibit its broad spectrum of activity against multi drug resistance microorganisms [7–10]. A quest for an environmentally sustainable synthesis process has led to a few biomimetic approaches like applying natal principles in the formation of nanoparticles. Among several, bioreduction is the prime and widely practiced functional method in synthesizing the nano materials [11, 12]. Noble metal nanoparticles such as silver, gold, which are geared up by plant extracts, algae, bacteria and fungi are broadly applied in drug delivery systems [13], electronics [14, 15], biosensors [16], cancer therapeutics and antimicrobial agents [17–19]. For three decades, exploration of marine algae has been far above the ground for the search of new and effective natural origin medicines, because it posses elevated quantity of concealed bioactive essence. Several of such compounds, including carbohydrates, alkaloids, steroids, phenols, saponins and flavonoids, etc. [20–22]. These multifaceted compounds exhibit a wide range of industrial and biotechnological applications [23]. An extent, these bio-molecules play decisive role in reduction of metal ions and generate the stable eco friendly nanoparticles. Further, AgNP’s fetches superior antibacterial activity by interacting with thiol clusters present in bacterial cell by cliping its replication. Literature strappingly supports that these Ag^+^ ions unyoke the respiratory chains and collapse the proton motive forces across the cytoplasmic membrane of bacteria [24]. *Gracilaria crassa* (*G. crassa*), a well-known red algae, having potential secondary metabolites [25]. In harmony to the above information, the present study was intended to prepare and typify silver nanoparticles from *G. crassa*, further to explore its antimicrobial activities against highly resistance microbial inhabitants.

## Methods

### Materials

Silver nitrate (AgNO_3_; Mol. Wt: 169.87; Prod. No: 27462) of analytical grade (AR) was purchased from Fisher scientific, Mumbai, India. The nutrient agar medium was purchased from Hi Media (Mumbai, India). All other chemicals used were analytical grade. Microorganisms such as *Escherichia coli* (*MTCC 443*), *Proteus mirabilis* (*MTCC 442*), *Bacillus subtilis* (*MTCC 441*) and *Pseudomonas aeruginosa* (*MTCC 424*) were obtained from Microbial Type Culture and Collection, Pune, India.

### Seaweed collection and extraction

*Gracilaria crassa* was collected along the coast of Mandapam (9°16′58.9″N 79°18′53.6″E), Rameswaram, Tamilnadu, India. The freshly collected algal material was rinsed with seawater followed by deionized water to get rid of extra impurities. The samples were kept in shade for 15 days drying. The algal material was ground to powder and uphold stockpile by placing at −4°C for further studies. The *G. crassa* extract was prepared in a conical flask by taking 2.5 g in 100 mL of deionized water. It was heated for 45 min at 50°C and positioned in an orbital shaker for 24 h in order to conquer the maximum extraction of compounds. The Extracts were filtered through whatman No. 1 filter paper and stored in refrigerator for further studies [26].

### Synthesis of silver nanoparticles

AgNP’s were synthesized by adopting the method proposed by Sathishkumar et al. with simple modification [27]. 5 ml of algal extract was added in to 95 ml of 1 mM aqueous silver nitrate solution, in 250 ml conical flasks and kept at 30°C in a shaker for overnight to facilitate absolute reduction. The samples were monitored periodically for its color intensity to confirm the formation of AgNP’s.

### Characterization of silver nanoparticles

#### UV–Vis spectral analysis

The reduction in pure silver ions was recorded by measuring the UV–Vis spectrum of the synthesized AgNP’s of *G. crassa* at room temperature with a Perkin Elmer Lambda 25 UV–Vis spectrometer at the wavelength of 200–800 nm [28].

#### Particle size and zeta potential studies

Particle size and zeta potential experiments for *G. crassa* AgNP’s were carried out by using a HORIBA Instruments (Singapore) Pvt Ltd, Singapore.

#### Powder x-ray diffraction (XRD) analysis

The silver nanoparticles were alienated by repeated centrifugation at 12,000 rpm for 10 min followed by redispersion of AgNP’s into deionized water for three times. The supernatant was transferred in microwave for drying. X-ray diffraction (XRD) measurement of the AgNP’s was carried out using Rigaku smart lab instrument (Japan), function at voltage of 40 kV, 30 mA current with Cu Kα1 radiations.

#### FESEM-EDAX studies

After careful UV–Vis spectroscopical analysis of synthesized nanoparticles, diameter of nanoparticles were further confirmed by Field emission scanning electron microscopy–energy dispersive X-ray analysis (FESEM–EDAX) by using SUPRA 55-CARL ZEISS, Germany.

#### Antibacterial assay

The antibacterial evaluation of AgNP’s was conceded by using different experimental pathogens like *Escherichia coli* (*MTCC 443*), *Proteus mirabilis* (*MTCC 442*), *Bacillus subtilis* (*MTCC 441*) and *Pseudomonas aeruginosa* (*MTCC 424*) maintained by department of Pharmaceutical Biotechnology, SVCP, Tirupati, India. Prior to experimentation, untainted cultures was subcultured into nutrient media. Nutrient agar plates were prepared, seeded and pierced with 20 and 40 µg/ml of AgNP’s. Streptomycin sulphate (20 µg/ml) was used as standard [29].

## Results and discussion

### UV–Vis spectral analysis

**Fig. 1:**
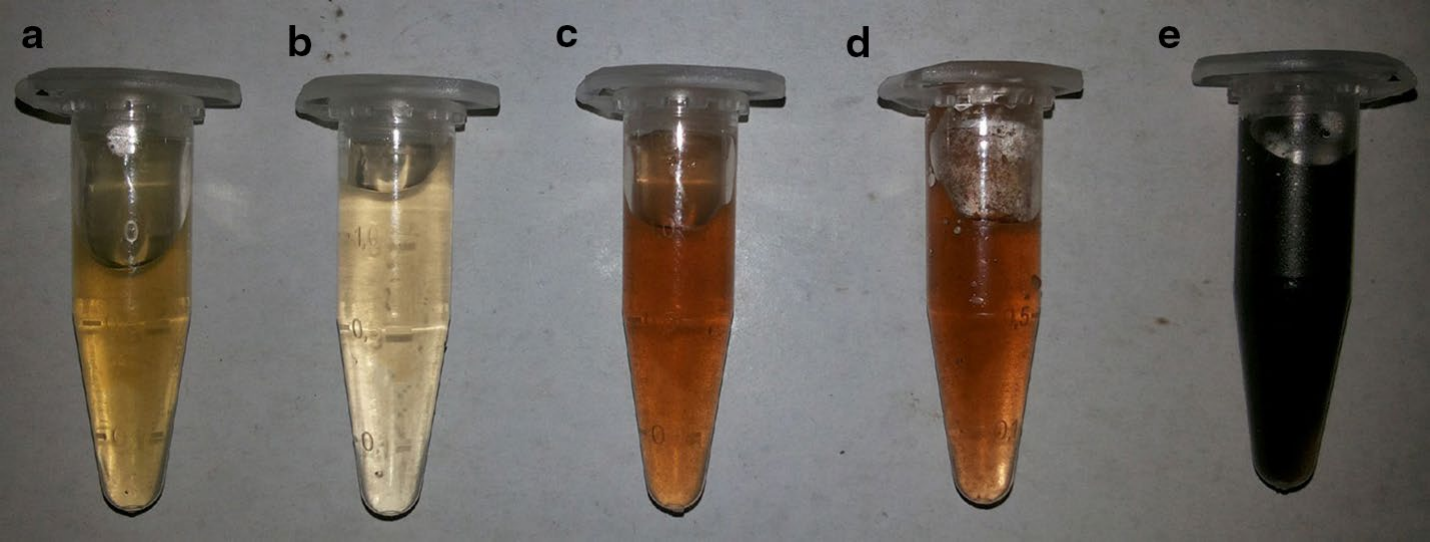
Formation of silver nanoparticles by green synthesis. AgNP’s were formed by the reduction of silver ions by *Gracilaria crassa*. This figure illustrates the various stages of formation of AgNP’s; **a** pure algal extracts (*pale yellow*); **b** at 0 min, on immediate addition of 1 mM solution of Silver nitrate (no reaction); **c** after 15 min (slight reduction); **d** after 30 min (moderate reduction); **e** after 12 h of addition of 1 mM solution of Silver nitrate (Complete reduction).

**Fig. 2:**
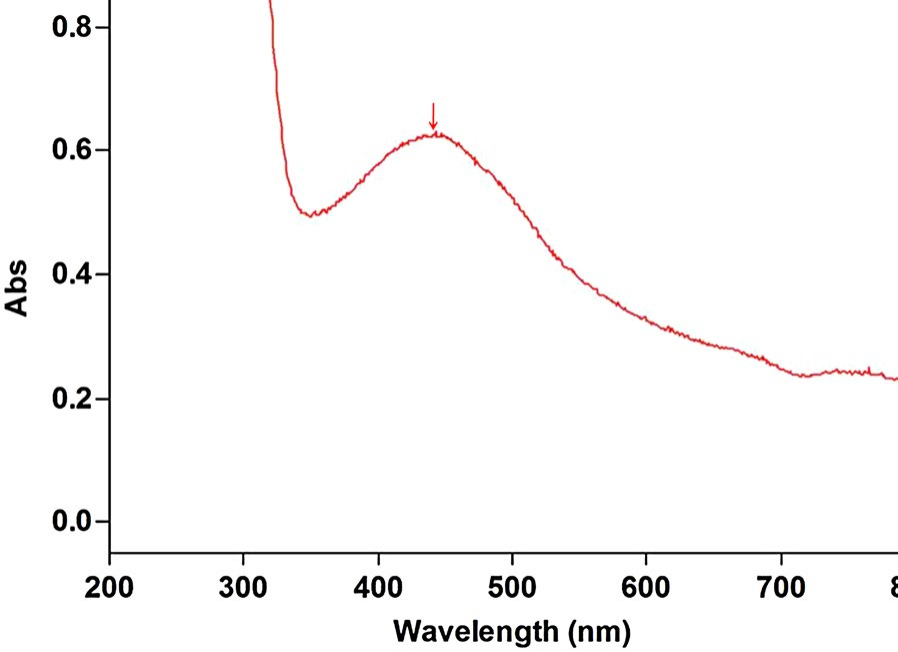
UV-Vis absorption maxima of silver nanoparticles. The data is based on the presence of absorbance peak of AgNP’s solution at the wavelength range of 400–450 nm. The absorption maxima were found to be 434 nm.

The formation of AgNP’s was defined by color transformation [30] from pale yellow to dark brown (Fig. 1). The color change is due to the excitation of the surface plasmon resonance (SPR) (Fig. 2) which elicits λ max of 443 nm.

### Particle size and zeta potential

**Fig. 3:**
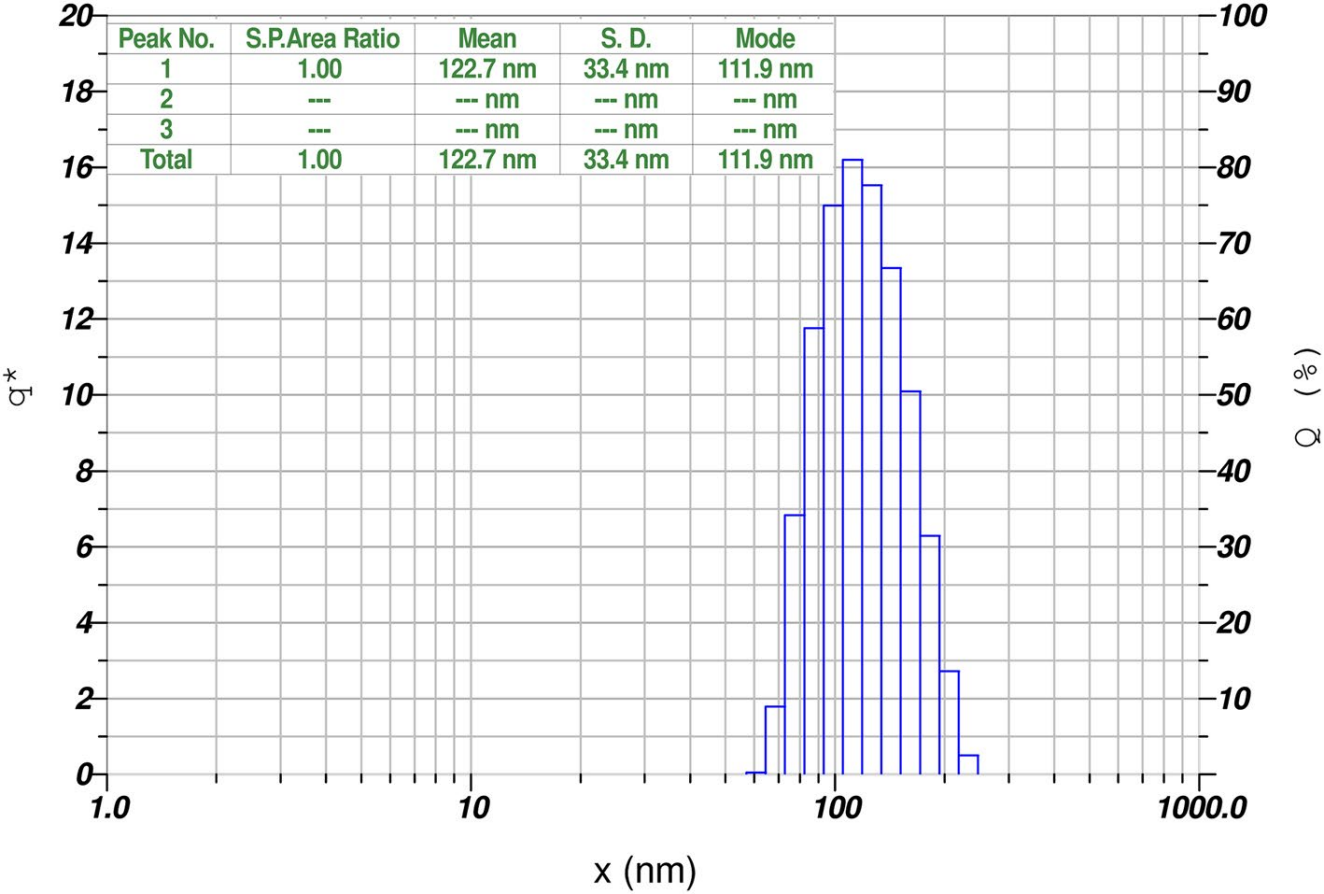
Particles size distribution of AgNP’s prepared from *G. crassa* extracts. Average particle size of synthesized AgNP’s ranges from 60 to 200 nm

**Fig. 4:**
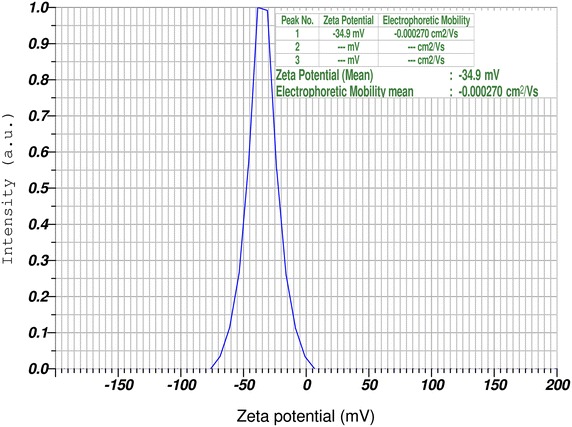
Zeta potential illustration of AgNP’s. Zeta potential distribution of synthesized AgNP’s prepared from *G. crassa* extracts.

Particle size determination of synthesized AgNP’s was revealed underneath by intensity. Laser diffractions exposed by obtained AgNP’s were in polydisperse concoction with average size of 122.7 nm (Fig. 3). The zeta potential endows stability of nanoparticles and surface charge. The zeta potential was found to be −34.9 mV. Earlier reports strongly supports, when the zeta potential value positions between −30 and −50 mV, it specifies good stability of nano particles [31]. The high negative value (Fig. 4) substantiates the repulsion between the particles and thereby increases the stability of the AgNP’s.

### X-ray diffraction (XRD) analysis

**Fig. 5:**
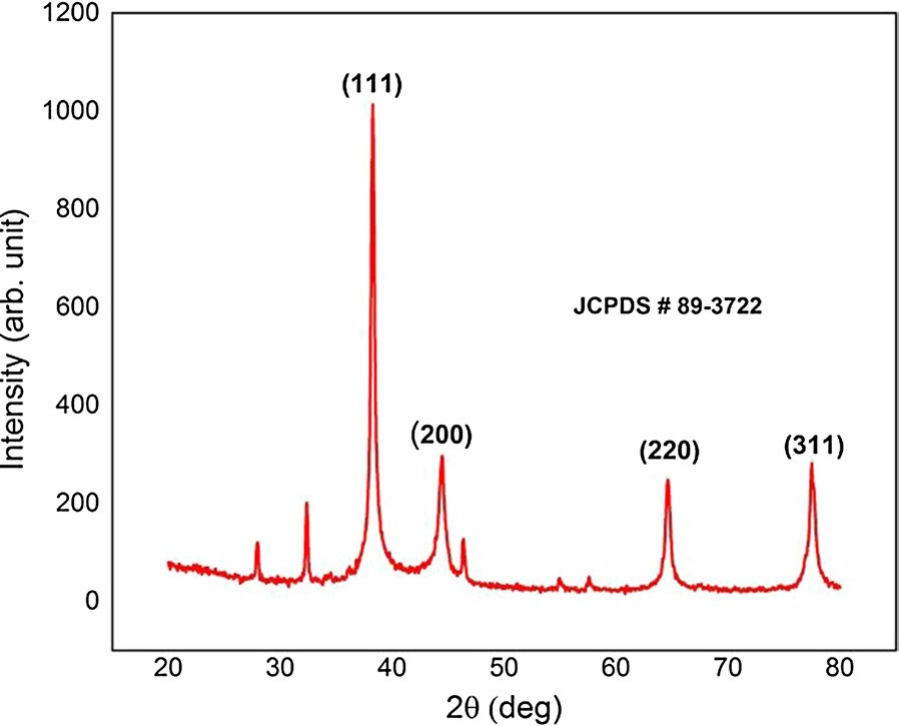
X-ray diffraction analysis of bio synthesized silver nanoparticles using marine red algae extracts of *G. crassa* corresponds four Bragg’s reflections planes of the metallic silver nanoparticles.

The XRD pattern of powder sample of *G. crassa* AgNP’s exhibited peaks at 38°, 44°, 64° and 77°. Four Bragg’s reflections corresponding to (111), (200), (220) and (311) planes of the fcc crystal structures of metallic silver (JCPDS No. 89-3722) are interpreted from the XRD (Fig. 5). The orientation (111) is more predominant since it shows high intense. Broadening of the diffraction crest disclose the formation of pure crystalline silver [32].

### FESEM and energy dispersive X-ray analysis

**Fig. 6:**
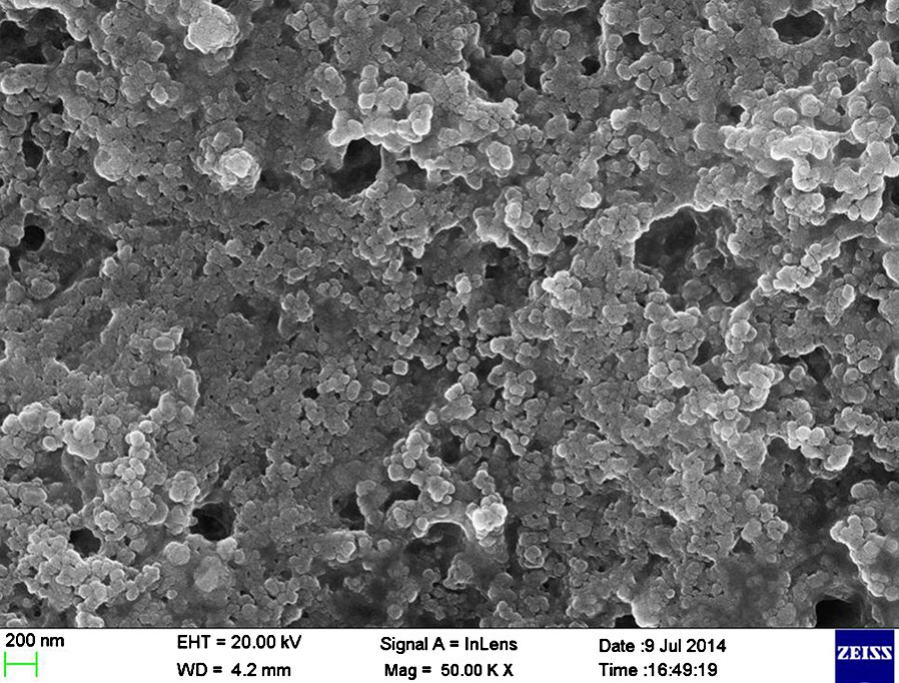
Morphological images of synthesized AgNP’s by using FESEM. This figure shows individual nanoparticles in clusters, spherical and some with irregular texture.

**Fig. 7:**
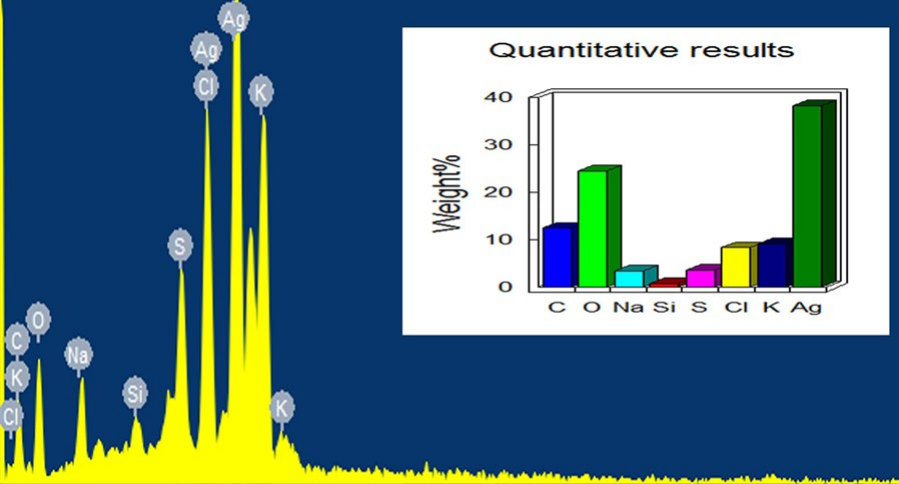
Energy dispersive X-ray analysis of silver nanoparticles. EDAX profile of AgNP’s shows higher percentage of silver signal.

Field emission scanning electron microscopy investigation was further confirmed the size of silver nanoparticles synthesized from *G. crassa*. The size (diameter) of the nanoparticles ranges between 60 and 200 nm and the shapes were spherical and some are irregular (Fig. 6). The outcome of FESEM reports were overlapped with earlier reports [33, 34]. The energy dispersive X-ray analysis (EDAX) depicts strong indication in the silver region, which authenticate formation of silver nanoparticles (Fig. 7). The optical absorption peak at 3 kV, which attributed to metallic silver nanocrystallites owing to surface plasmon resonance [35, 36].

### Antibacterial assay

**Fig. 8:**
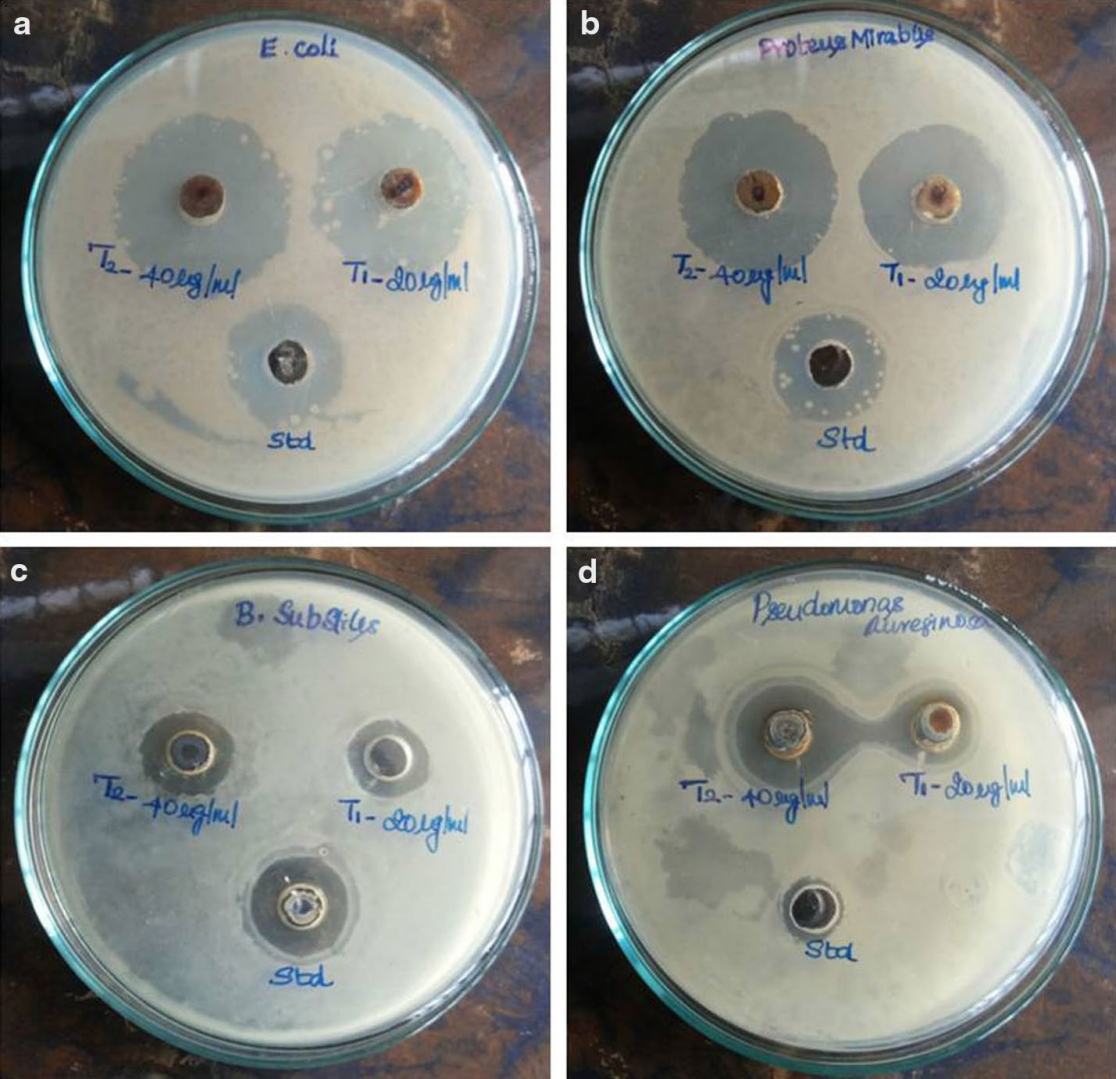
Antibacterial activity of AgNP’s. Zone of inhibition of silver nanoparticles against **a** *Escherichia coli*, **b** *Proteus mirabilis*, **c** *Bacillus subtilis*, **d** *Pseudomonas aeruginosa*; [T_1-_(20 µg/ml of AgNP’s) and T_2_ (40 µg/ml of AgNP’s); Standard (Std-Streptomycin sulphate 20 µg/ml)].

**Fig. 9:**
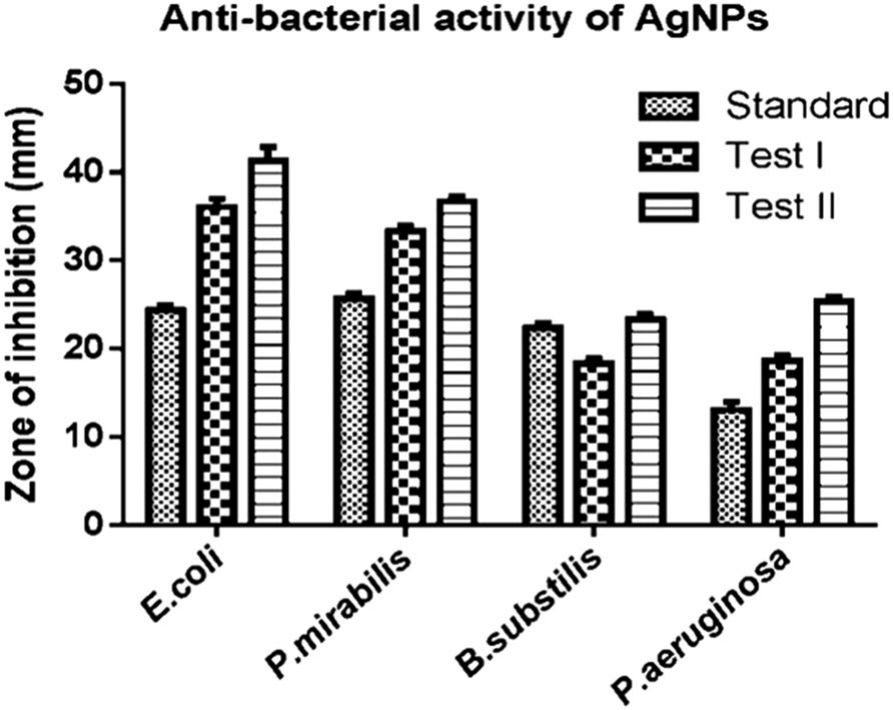
Sensitivity prototypes of silver nanoparticles against various microbial pathogens.

Antibacterial activity by agar well diffusion technique was recorded after 24 h incubation of culture plates. The AgNP’s demonstrate excellent antibacterial activity against all tested microorganisms (Fig. 8). AgNP’s showed high spectrum of activity against *E. coli* and *P. mirabilis* at the concentrations of 20 and 40 µg/ml when compared with standard (Fig. 9). The significant zone of inhibition was exerted due to effect of AgNP’s on biochemical process of the bacterial cell by interacting thiol and amino groups of proteins and nucleic acids of cell wall [37–39]. Further, this could lead to interaction between nanoparticles and microorganism which results in triggering the discharge of highly reactive oxygen species (ROS), mostly hydroxyl radicals and singlet oxygen [40–42]. This augments the deposition of nanoparticles on the bacterial cell surface results large accumulation of silver nanoparticles causing disruption of cellular functions.

## Conclusion

In summary, the bio-reduction of aqueous silver ions to silver nanoparticles (AgNP’s) was successfully done using marine red algae, *G. crassa*  in trouble-free, economy and ecofriendly manner. The average size of silver nanoparticles was found to be 122.7 nm with high stability of −34.9 mV. Further characterization by UV–Vis spectroscopy, FESEM, EDAX confirm formation nanoparticles which are virtually spherical in shape. XRD divulges fcc structural confirmation. The proved antibacterial potential will lend a hand to develop a powerful antibacterial formulation in near future as biomedical remedies. Hence, authors robustly propose this green synthesis of nanoparticles can be extended to the wide range of applications.

## Abbreviations

AgNP’s: silver nanoparticles
FESEM: field emission scanning electron microscopy
EDAX: energy dispersive analysis of X-rays
XRD: X-ray diffraction
Fcc: face centered cubic
Cu: copper
mV: millivolts
SPR: surface plasmon resonance
kV: kilovolts
mM: millimolar
nm: nanometer
ROS: reactive oxygen species
MTCC: microbial type culture and collection
